# Linking the long-term variability in global wave energy to swell climate and redefining suitable coasts for energy exploitation

**DOI:** 10.1038/s41598-022-18935-w

**Published:** 2022-08-29

**Authors:** Bahareh Kamranzad, Khalid Amarouche, Adem Akpinar

**Affiliations:** 1 Hakubi Center for Advanced Research, Kyoto University, Kyoto, 606-8501 Japan; 2 Graduate School of Advanced Integrated Studies in Human Survivability (GSAIS), Kyoto University, Kyoto, 606-8306 Japan; 3 Department of Physics, Faculty of Natural Sciences, Imperial College London, London, SW7 2AZ UK; 4 Department of Civil Engineering, Bursa Uludağ University, 16059 Bursa, Turkey

**Keywords:** Physical oceanography, Civil engineering

## Abstract

The sustainability of wave energy linked to the intra- and inter-annual variability in wave climate is crucial in wave resource assessment. In this study, we quantify the dependency of stability of wave energy flux (power) on long-term variability of wind and wave climate to detect a relationship between them. We used six decades of re-analysis wind and simulated wave climate in the entire globe and using two 30-yearly periods, we showed that not only the previously suggested minimum period of 10 years for wave energy assessment appears to be insufficient for detecting the influence of climate variability, but also the selection period for wave energy assessment can lead to an over/underestimation of about 25% for wave power. In addition, we quantified the dependency of rates of change of wave power, wind speed and wave parameters and showed that the change in wave power is mainly a function of change in swell wave climate globally. Finally, we redefined the suitability of global hotspots for wave energy extraction using intra-annual fluctuation, long-term change, and the available wave power for the period of six decades. The results highlight the importance of climate variability in resource assessment, sustainability, and prioritizing the hotspots for future development.

## Introduction

Energy crisis and climate change are the consequences of using fossil fuels. While the world’s population is expanding, providing energy becomes more critical considering the limited resources. Renewable energies -including hydropower, wind, solar, bioenergy, geothermal and marine- have been developing to tackle the negative impacts of climate change and provide energy supply. Areas in the vicinity of oceans and seas can utilize unlimited ocean resources such as offshore wind and marine energy (including wave, tidal, current, and salinity gradient energy (or so-called blue energy), as well as ocean thermal energy conversion (OTEC)) to provide a portion of energy demand.

Although the contribution of wind energy to renewable energy supply has been around 25.06%, the ratio of global cumulative installed electricity capacity of offshore to onshore wind has been only 4.92% in 2020^1^. On the other hand, marine energy has contributed only to 0.02% (526.843 MW) of the total cumulative installed electricity capacity of all renewables (2.92 TW) in 2020^1^. Wave energy with the highest density within all ocean renewables can contribute to the diversity of the renewable energy mix with additional benefits of being predictable and endless, having lower visual and environmental impacts, and broad geographic viability. In addition, it can be used for other purposes such as desalination, hydrogen production, pumping and heating processes, and coastal protection by reducing coastal erosion^2^. The floating wave energy farms have the advantage of being adaptable to sea-level rise (SLR), as well.

Despite the numerous advantages of wave energy, it is highly affected by climate fluctuation since gravity waves are generated by wind, which is highly affected by climatic fluctuations^3^. Long-term trends in wind and wave climate and consequently wave energy might be affected by natural phenomena and ocean teleconnection patterns such as El Niño Southern Oscillation (ENSO)^4^, North Atlantic Oscillation (NAO)^5–7^, etc. Hence, recent studies have shown that assessing the sustainability of wave energy in terms of intra-annual variation and long-term changes has a key role in selecting suitable locations for the installation of wave farms^8,9^.

Change in wind and wave climate affect not only available energy resources but also the intensity and frequency of coastal disasters such as flooding and erosion -which may intensify the vulnerability of coastal regions to SLR^10^, the vulnerability of nearshore ecosystems^11^, and human activities such as navigation, transportation, and any design in the marine area^12^. Hence, it is necessary to investigate the offshore/coastal climate variability for future planning, prevention, and mitigation of natural disasters^13,14^. Furthermore, assessing the intra-annual variability in wind and wave climate can be used for zone classification of a certain domain^15–19^, whereas the long-term change is also necessary for defining the stability and sustainability of wave climate and energy^8,9,20–23^. Although wave energy assessment has been suggested by the International Electrotechnical Commission (IEC)^24^ to be performed for a minimum of 10 years, long-term variability of wind and wave climate is mainly investigated using a time span of about 30 years in order to reduce the uncertainties associated with climate variability^25^. For instance, some climate patterns such as Interdecadal Pacific Oscillation (IPO), Pacific Decadal Oscillation (PDO), or Southern Annular Mode (SAM) have a low frequency of variability that might not be accounted for when analyzing only one decade^26–28^.

There are several studies on assessing the long-term trend of wind and wave climate on global, regional, and local scales. A recent study based on a re-analysis wave hindcast has shown a global increase of 0.4% per year in global wave power since 1948 as a consequence of oceanic warming^29^. Satellite altimetry data of 23 years have also shown an increase in both wind speed and wave height^30^. Assessment of global satellite data over the period from 1985 to 2018 has shown slight increases in wind speed and wave height with larger increases in extreme conditions, mainly in the Southern Ocean^31^. Trends in ocean surface waves over 1992–2017 have been assessed using different sources, including altimetry products and two reanalysis and hindcast datasets, showing general similarity in spatial variation for different resources in most areas^32^. The ability of multi-mission altimeter datasets to detect trends in long-term significant wave height has also been investigated, and the results have shown an accuracy of ± 0.2 cm/year for measuring the trends in mean and 90th percentile significant wave height^33^. In the north Atlantic, significant changes in winter extremes of H_s_ related to NAO have been detected using a 40 yearly global wave hindcast^34^. In addition, in the northeast Atlantic, wave height trend has been found significantly increasing at northern latitudes with the less significant increasing trend for peak period according to 57 years of wave hindcast^5^. In North Pacific, an increasing trend of wave power, except for the more recent periods, has been observed using 60 years of wave hindcast^4^. The recent decrease in wave power across much of the North Pacific has been understood to be associated with the prevalent Pacific Decadal Oscillation (PDO) cool phase that developed after the late 1990s^4^.

On a regional scale, the long-term trend of wave energy around Japan has shown variable trends in different areas, with an increase in the southern half of the east coast of Japan using 30 years of in-situ wave measurements^35^. In contrast, a more recent study has shown a decrease in wave power over 55 years using a re-analysis dataset, especially on the southern coasts of Japan^22^. Both satellite and re-analysis data have shown an increase in mean wave height in China's coastal seas^36^. Additional studies in the China Sea using a 24-yearly wind and wave climate assessment have also reported a significant increase in both wind and wave in the whole domain with some notable regional differences^12,14^. A 31-yearly wind and wave climate assessment in the South China Sea has revealed various wind and wave parameters trends in different months^37^. A recent study based on 55 years of wave simulation has identified a significant decrease in wave energy in the eastern parts of the South China Sea^9^. In southwest Western Australia, anomalies in wave heights related to the shift of the Southern Ocean storm belt and the subtropical high-pressure ridge have been reported using a 21-yearly wave hindcast^38^. A declining trend in the mean and extreme wind speed has been found in the Arabian Sea and Bay of Bengal (BoB), while an increasing trend in extreme wave height has been detected there using 33 years of wind and wave climate^39^. Another study in the BoB has also disclosed an increasing trend of wave height using monthly averaged satellite and hindcast wave data for 20 years^40^. A slightly positive trend of 0–0.2% per year in H_s_ in the Persian Gulf has been found to be associated with positive trends in wind speed since the area is dominated by seas due to the inadequate fetch length that limits swell development^41^. In the Red Sea, a long-term decline in wave height has been detected based on a 30-yearly hindcast^42^. In the Black Sea, no noticeable trend in H_s_ and WS has been revealed based on a 31-yearly wave hindcast except for a few locations where a weak increasing trend in mean WS along the north-eastern coasts of Turkey and the Crimean peninsula, and a weak decreasing trend in mean H_s_ along the north-western coasts of Turkey has been found^43^. The mean wave power has also shown a decreasing trend in the hotspot areas of the Black Sea according to 31 years of wave hindcast^44^. A decrease in mean and extreme wave climate as well as the average intensity of extreme events has been found in the Hellenic Seas based a 42-yearly analysis of wave hindcast^45^. However, a significant increasing trend in annual maximum of wave height and wind speed and storm wave intensity has been represented in the western Mediterranean based on 41 years of re-analysis wind and wave data^46,47^. In addition, no substantial long-term change has been seen in wind and wave climate in the North Sea based on 56 years of wave hindcast and re-analysis wind data^48^.

The above-mentioned studies highlight the importance of assessing the links between the trend and long-term changes in wind and wave climatology and the wave energy resources and their sustainability. However, the relationship between the change of parameters and its dependency on the length of time series with regards to their spatial variation remains indistinct and unquantified. Moreover, the obtained results highly depend on the choice of the assessment period, which depends on the length of the available data. Therefore, in the present study, we focus on finding a relationship between the change of wind and wave characteristics on a global scale and investigate the dominance of wave climate in various regions and whether it changes with time. For this purpose, we use six decades of re-analysis wind field and modeled wave data.

Our previous study^22^ has shown that it is necessary to consider the long-term change in wave energy resource assessment and has proposed the climate-dependent sustainability criteria^22^. Accordingly, we assess the relationship between the change in wave power and change in wind and wave climate, spatially and temporally, to determine whether the change in available wave energy is predictable. Considering the wind and wave climate variability, such a relationship might be different in different parts of the globe. In addition, the relationship can change over time. Moreover, swells play an important role in long-term changes in coastal morphology and offshore hazards^49^. However, their role and dominance in wave energy variation have not been significantly investigated. Hence, in the present study, we also evaluate the wave energy dependency to swell climate particularly along with the wind and wave climate. Finally, we utilize the long-term in available resources to redefine the suitable coasts for wave energy exploitation aligned with Sustainable Development Goals (SDGs).

## Results and discussion

Various studies use different time slices for estimation and spatio-temporal assessment of the wind and wave climate and resource, which is typically a period of 30 years^25^. However, as mentioned before, the results might be sensitive to the selection of the assessment period. In order to discuss the importance of selecting the suitable period for wind and wave climate assessment, we first divide the period of data availability into two periods of 30 years each (1960–1989 and 1990–2019) to investigate the impact of the selected period on the results. Later, we continue with the decadal analysis of the wind and wave climate and resources to discuss the relationship between the change of different parameters in more detail and show how the change of parameters in long-term is a dependent of sea/swell domination.

### Change in the 30-yearly mean annual wind and wave characteristics

**Figure 1:**
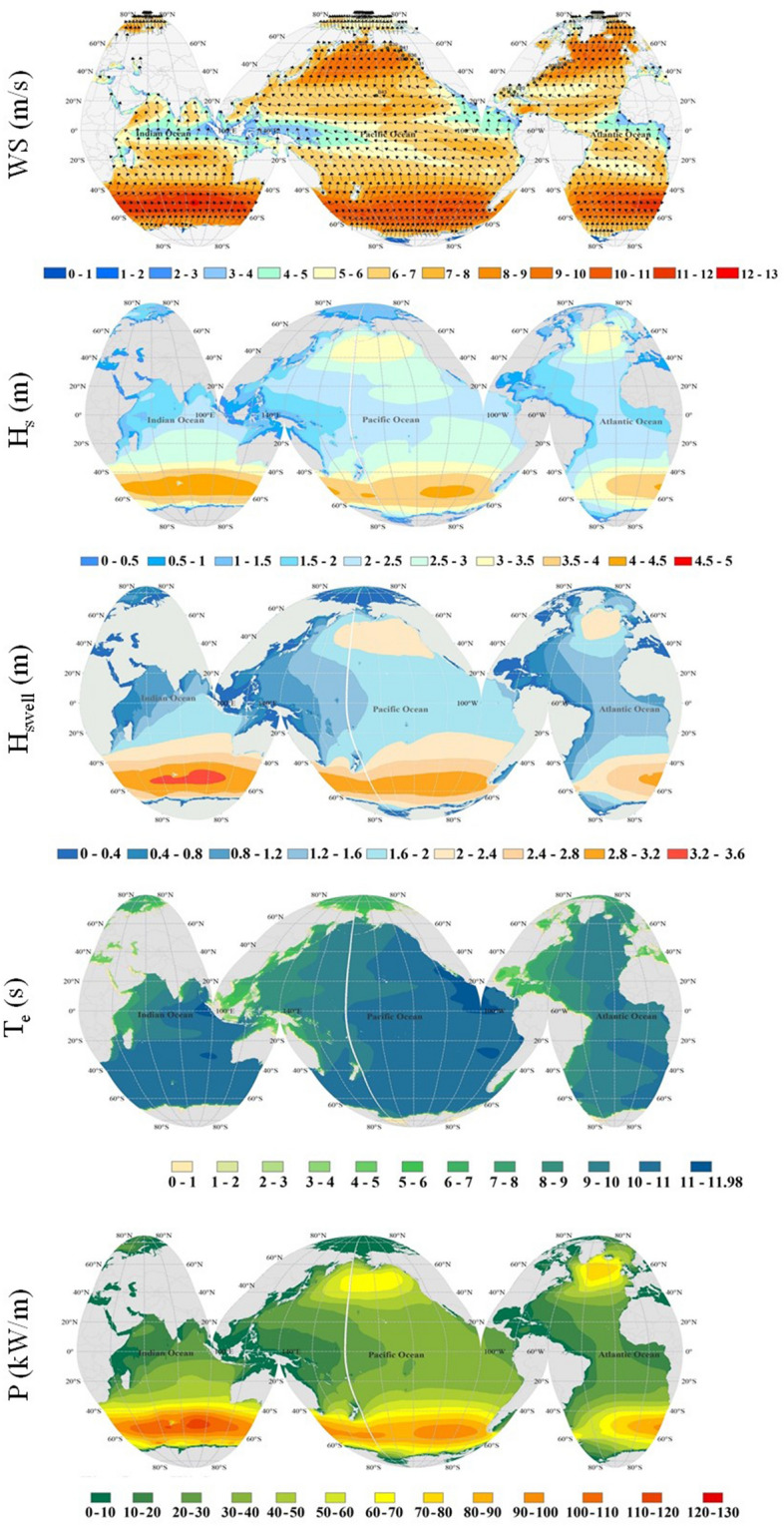
Annual mean values of different parameters in Per_1. The figure has been generated using ArcGIS 10.2 and Natural Earth-Free vector and raster map data @ naturalearthdata.com.

**Figure 2:**
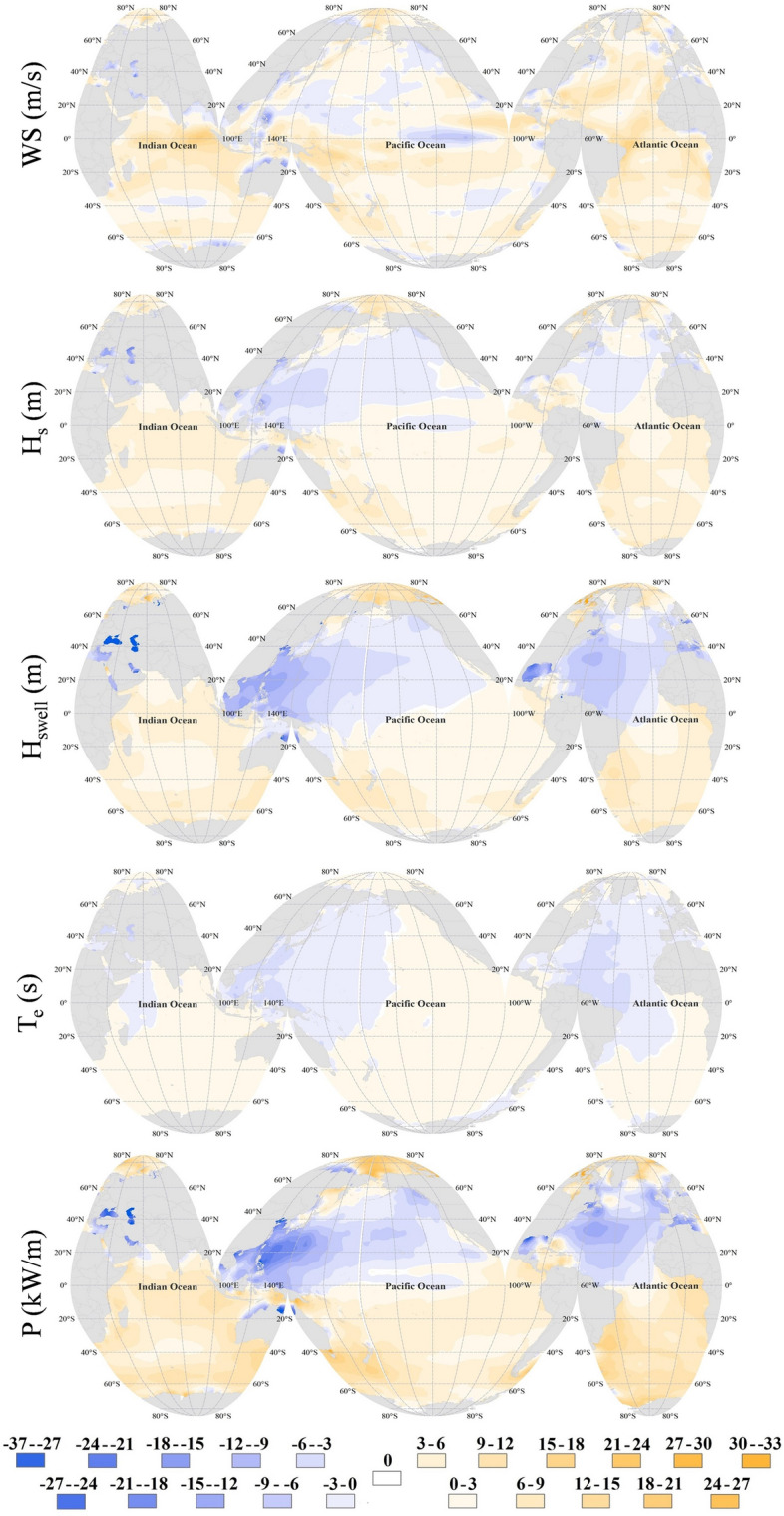
Relative change of annual mean values of different parameters in Per_2 compared to Per_1 (%). The figure has been generated using ArcGIS 10.2 and Natural Earth-Free vector and raster map data @ naturalearthdata.com.

We used the 60 years of the wind field and simulated wave characteristics to assess the change of mean values in two long-term (i.e., 30-yearly) periods; Per_1: 1960–1989, and Per_2: 1990–2019. Figure 1 shows the mean values of different parameters, including wind speed (WS), H_s_, swell wave height (H_swell_), and wave power (P) in Per_1, whereas Fig. 2 shows the relative change of such parameters in Per_2 compared to Per_1. The relative change has been calculated based on the ratio of the difference between Per_2 and Per_1 to Per_1. Investigating the spatial distribution of wind and wave climate over the globe in the first period (Per_1), we find the severe wind and wave climate, i.e., higher WS, H_s_, H_swell,_ and P in the southern hemisphere due to westerlies. The severity declines when approaching the equator under the tropical easterlies or trade winds and increases again with the westerlies in the northern hemisphere.

The relative change in wind and wave characteristics (Fig. 2) shows a different spatial pattern of change in wind speed and wave characteristics. A previous study^50^ based on 26 years of corrected satellite altimetry data has also shown that the wind speed and wave height trend can be different, which might be due to the domination of swells rather than locally generated waves. Another regional study^39^ in the Arabian Sea and Bay of Bengal has also confirmed that the conflicting trend in wind and wave climate is mainly due to the swell dominance in the region. In addition, another study showed that the wave power attributed to swells is stable over the long term as swells remain unchanged in Sri Lanka -where the wave climate is dominated by swells from the Southern Ocean^21^- according to the future projections of wave climate there^51^.

Figure 2 shows a dominant decrease in the wave characteristics in the northern hemisphere, except for the northern Indian Ocean during the second (more recent) period. However, the wave energy period (T_e_) also has a slight decrease in the northern Indian Ocean. The significant decreases in P in areas such as the northern Atlantic, Gulf of Mexico, and eastern Pacific seem to be connected to the decrease in H_swell_, which is also shown by the decrease of wave period in those regions. The reduction in H_swell_ in the western Pacific seems to be due to the reduction in the wind speed by easterlies in the northern Pacific^22^, which are the dominant winds generating swells propagating to the west.

The increase in the wind speed, wave and swell heights, and wave power is seen in the southern hemisphere during the second period (Fig. 2). However, the increase in wave power has been more considerable in the southern Atlantic and southwestern Pacific, where it reaches to around 25% in areas such as southeast Australia and the east coasts of South America. The reason for larger decrease in P compared to H_s_, H_swell_ or T_e_ is related to wave power estimation formula which indicates that the wave power is proportional to the square of the wave height multiplied by the wave period. The results are similar to those of Reguero et al.^29^ based on 24 years of wave power estimation. The results also reveal that selecting a different assessment period in this study has lead to an estimated error of up to around ± 15% in WS, ± 10% in H_s_, ± 18% in H_swell_, ± 3% in T_e_ and ± 25% in P in the open ocean.

### Decadal variation of mean annual wind and wave characteristics

The previous section showed that the change of wave power spatially follows the change of swell wave height. However, previous studies on wind and wave climate variability have shown that long-term trends in wind and wave climate might not be monotonic^10,45,52^, and hence trend evaluation in shorter time slices might be necessary. In addition, changes in decadal wave energy might show different relationships with wind and wave parameters such as WS and H_s_^9^. Hence, in this section, we break the sixty years of wind and wave data to study the decadal variability of wind and wave climate. In order to investigate the relationship between the change in wave power and swell wave height on a decadal scale, the whole period of the simulation was divided as Dec_1: 1960–1969, Dec_2: 1970–1979, Dec_3: 1980–1989, Dec_4: 1990–1999, Dec_5: 2000–2009, and Dec_6: 2010–2019.

**Figure 3:**
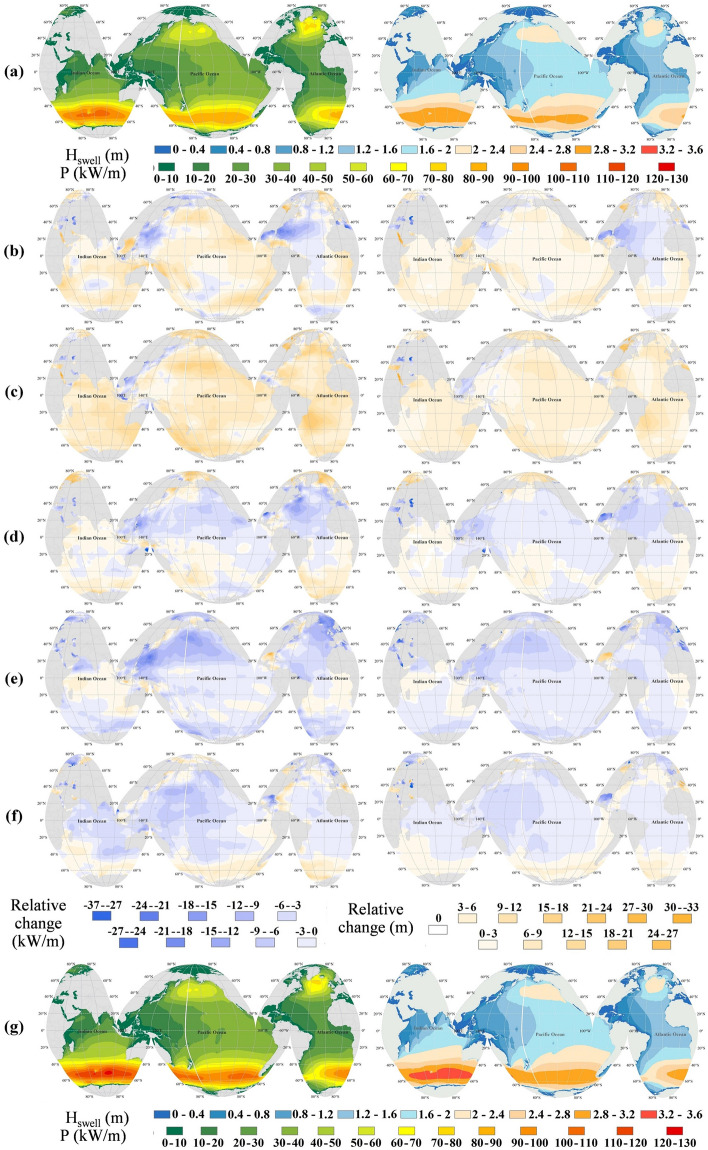
(**a**) Mean annual values in Dec_1, relative change in mean annual values in (**b**) Dec_2 compared to Dec_1, (**c**) Dec_3 compared to Dec_2, (**d**) Dec_4 compared to Dec_3, (**e**) Dec_5 compared to Dec_4, (**f**) Dec_6 compared to Dec_5, (**g**) Mean annual values in Dec_6. Left panel: P (mean values (**a** and **g**) in kW/m, relative changes in %). Right panel: H_swell_ (mean values (**a** and **g**) in m, relative changes in %). The figure has been generated using ArcGIS 10.2 and Natural Earth-Free vector and raster map data @ naturalearthdata.com.

Figure 3 indicates the mean annual values of P and H_swell_ in Dec_1, their decadal change in the following decades, and the annual mean in Dec_6. The left panels are for P, whereas the right panels are for H_swell_. According to Fig. 3, the change in P and H_swell_ has been different in various decades. Compared to Dec_1, P has generally increased in the northern Pacific, around the equator, and the Southern Ocean but decreased in the northern Atlantic and the northern Indian Ocean in Dec_2. The largest decadal decrease in mean P in Dec_2 is found to be around 20% in the Gulf of Mexico, which corresponds to a decrease of around 15% in H_swell_. In Dec_3, both P and H_swell_ have increased in the whole globe compared to Dec_2 (Fig. 3d), except for east and southeast Asia. The largest increase in P (around 25%) and H_swell_ (around 15%) can be found in the north Pacific and eastern coasts of South America. After such an increase in Dec_3, Dec_4 shows a slight decrease in wave power and swell height, mainly in the northern hemisphere, while such decrease in both parameters has become more intense in Dec_5, especially in the northern hemisphere where the amount of change reaches around 25% and 15% for the P and H_swell_, respectively. Moreover, most parts of the globe have experienced a slight decrease in Dec_6 compared to Dec_5 except for the Southern Ocean and the stripe between 40° S and 60° S, where southern westerlies are dominant, with a slight increase of about 10% and 5% in P and H_swell_, respectively. Comparison between Dec_1 and Dec_6 (Fig. 3a and g) show that in 50 years, despite spatially variable decadal fluctuation of the swell height and wave power, they have mainly changed in the southern hemisphere and especially where the southern westerlies are dominant (40° S–60° S). This means that the swells have become higher in 40° S–60° S in recent decades.

### Relationship between decadal rates of changes of different parameters

**Figure 4:**
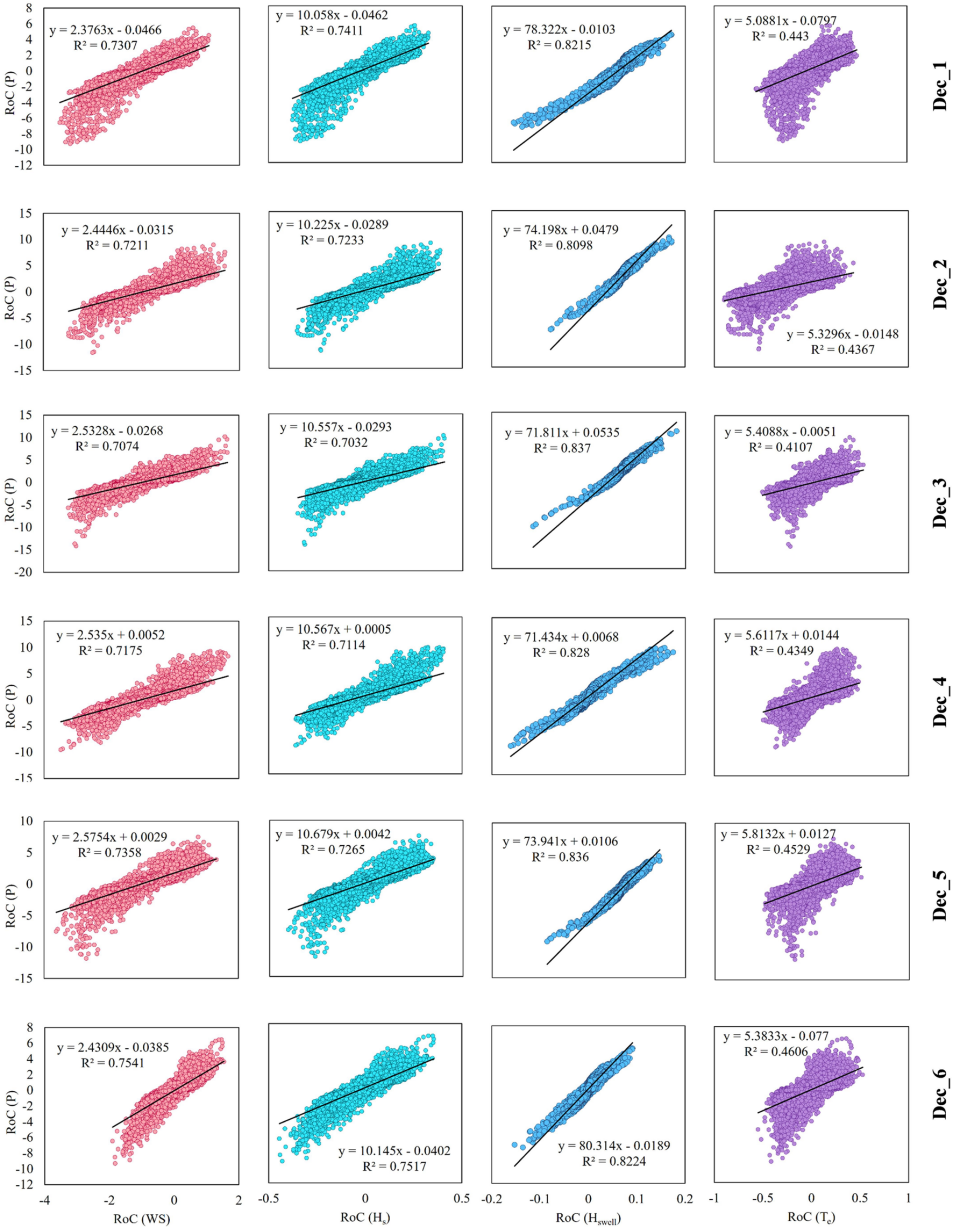
Rows from top to bottom show Dec_1 to Dec_6, and columns from left to right show linear regression between the rate of change of P and WS, H_s_, H_swell,_ and T_e_, respectively. The vertical axis shows the rate of change (RoC) of P, whereas the horizontal axis shows the RoC for other wave parameters.

The previous sections indicated that the spatial distribution of change of swell wave height and wave power are nearly similar across the globe. In this section the relationship between the change of wave power and various parameters is quantified. For this purpose, linear regressions between the decadal rate of change (RoC) of different parameters have been obtained for each grid point (42,328 points in total in the whole globe) and are depicted in Fig. 4. The RoC values have been obtained based on the slope of the best linear fit to the whole time series of each parameter for each decade and with a temporal resolution of 6 h (for 14,608 or 14,612 time steps, depending on the decade and leap years).

The formulas on each panel show the linear fit to the rate of change of P as a function of the rate of change of wind or wave characteristics. The values of R^2^ shown in each panel is the square of the correlation coefficient (R) and calculated as Eq. (1):

1 $$ {\text{R}}\;{{ = }}\;\frac{{\mathop \sum \nolimits_{{\text{i}}} {\text{((x}}_{{\text{i}}}  - {{\bar{\rm x})}} \times {\text{(y}}_{{\text{i}}}  - {{\bar{\rm y})}}}}{{\sqrt {\mathop \sum \nolimits_{{\text{i}}} {\text{(x}}_{{\text{i}}}  - {{\bar{\rm x})}}^{{{2}}} \mathop \sum \nolimits_{{\text{i}}} {\text{(x}}_{{\text{i}}}  - {{\bar{\rm x})}}^{{{2}}} } }} $$

in which *x* and *y* are the rate of change of P and any wind or wave parameter (including WS, H_s_, H_swell_ and T_e_), respectively, and $\stackrel{\mathrm{-}}{\text{x}}$ and $\stackrel{\mathrm{-}}{\text{y}}$ are their average values, respectively.

Figure 4 shows a clear and direct relationship between RoCs of P and H_swell_ in all decades, with the highest correlation in Dec_3 and Dec_5. Interestingly, the correlation between the RoC of P and RoCs of WS and H_s_ are nearly similar. However, the linear regression slope is different for the two parameters. In addition, the RoC of P has the least correlation with RoC of T_e_. The correlation between the RoC of P and RoC of WS, H_s,_ and T_e_ has reached the highest in Dec_6. Figure 4 shows that the relationship between the change of P and the change of WS, H_s,_ and T_e_ is not straightforward. However, the relationship between the change in wave power and changes in swell heights seems more direct. This means that the long-term change of wave power is directly affected by the change in swell climate rather than the significant wave height in almost the entire globe.

**Figure 5:**
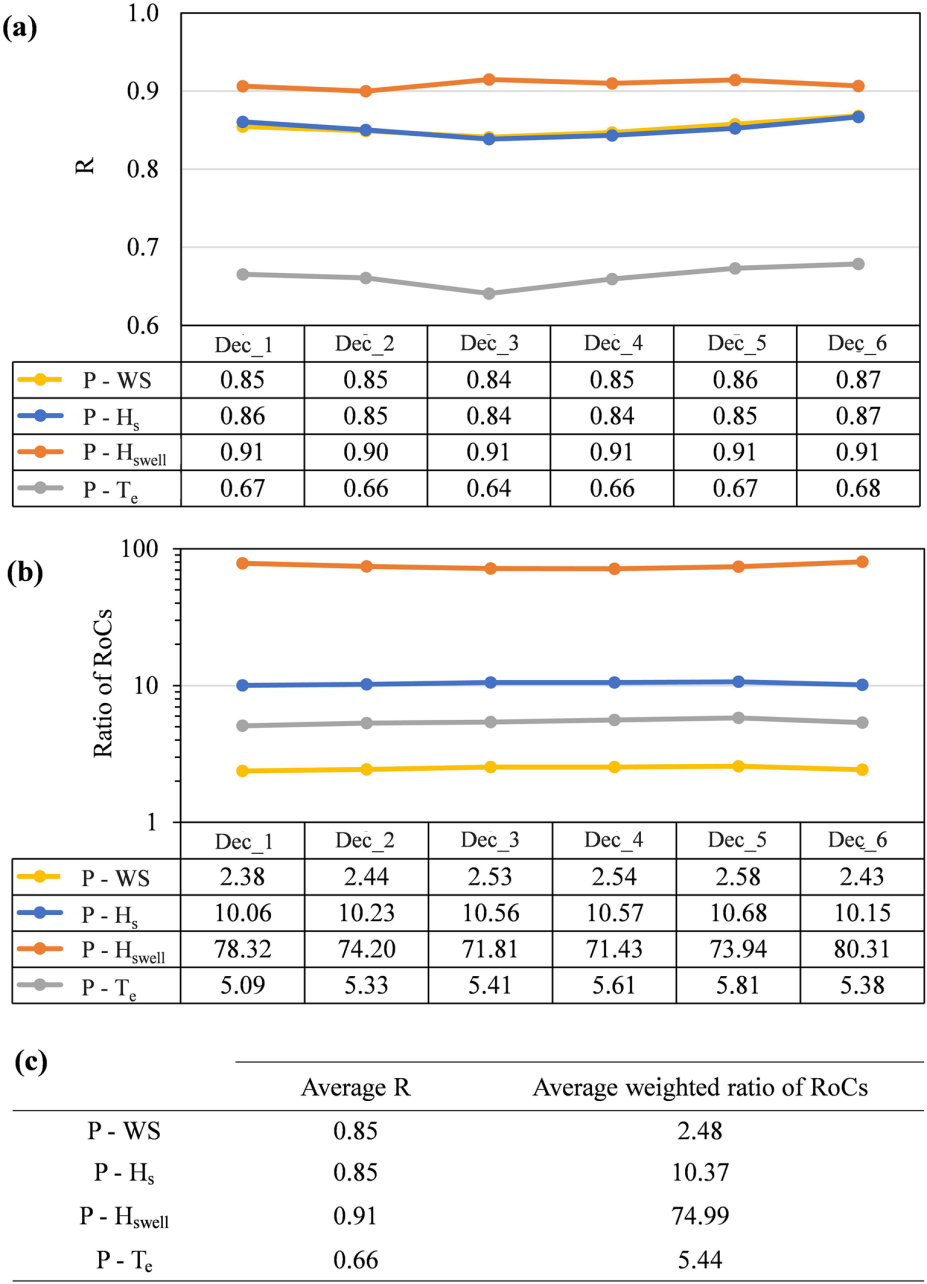
Decadal variation of (**a**) correlation coefficient (R) of RoCs and (**b**) ratio of RoCs, for P and wind or wave parameters and (**c**) the average values.

Figure 5 represents the summary of correlation coefficients for different decades and between the RoC of wave power and that of different parameters (Fig. 5a), as well as the ratio of RoC, which is the slope of the linear regressions in Fig. 4 (Fig. 5b). This figure again emphasizes that the wave power change is equally affected by the change in WS and H_s_ (based on similar R values in Fig. 5a). In addition, the decade with a higher correlation for RoC of P and H_swell_ (Dec_3) is the one with the lowest correlation between the RoC of P and T_e_. The ratio of RoCs (Fig. 5b) seems to remain nearly similar in all six decades, and hence, the weighted arithmetic means the ratio of RoCs has been calculated and shown in Fig. 5c with decadal R^2^ values as weights.

2 $$ {\text{Weighted}}\;{\text{ arithmetic }}\;{\text{mean}} = \frac{{\mathop \sum \nolimits_{{\text{i = 1}}}^{{\text{i = 6}}} \left( {{\text{Ratio \,of\, RoCs}}_{{\text{i}}} {{ \times {\rm R}}}_{{\text{i}}}^{{2}} } \right)}}{{\mathop \sum \nolimits_{{\text{i = 1}}}^{{\text{i = 6}}} \left( {{\text{R}}_{{\text{i}}}^{{2}} } \right)}} $$

where i demonstrates the decades and ranges from 1 to 6, implying Dec_1 to Dec_6. According to the calculated weighted ratio of RoCs, the RoC of P is almost 2.5, 10.4, 75, and 5.4 times the RoC of WS, H_s_, H_swell,_ and T_e_, respectively. Considering the correlation coefficients as a measure for the accuracy of the estimations for the relationship between RoCs, it can be concluded that RoC of P has been ~ 75 times the RoC of H_swell_ with 91% accuracy in the entire domain. It means that using the change in swell climate, we will be able to estimate the change in available global wave power with 91% accuracy. Furthermore, the correlation between the RoC of both WS and H_s_ is similar, which means that accepting the accuracy of 85% for predicting the wave power trend, only the wind speed trend can be sufficient.

### Priority coasts considering the variation and change in resources

The results in the previous sections showed that the long-term change in wave climate and energy could be considerable depending on the assessment period. Hence, in addition to intra-annual fluctuations, long-term changes in RoCs were considered to determine the stability of wave energy resources around the globe. For this purpose, Sustainability Index (SI_p_)^22^ was calculated in all output grid points and based on 60 years of simulated wave power (Eq. 2):

3 $$ {\text{SI}}_{{\text{p}}} = \frac{{\frac{{{\text{P}}_{{{\text{ave}}}} }}{{{\text{max(P}}_{{{\text{ave}}}} {) }}}{{ \times {\rm cos} }}\left( {\frac{{{\text{RoC}}}}{{{\text{max }}\left( {\left| {{\text{RoC}}} \right|} \right)}}} \right)}}{{{\text{MVI}}}} $$

in which, P_ave_ is the annual mean wave power for the period of 60 years, and max (P_ave_) is the maximum amount of P in the entire domain. MVI is the Monthly Variability Index, which is calculated based on the ratio of the difference between the highest and lowest monthly averages and the annual average in each grid point. The highest values of SI_p_ show higher mean wave power, lower monthly variability, and RoC, i.e., lower intra-annual variation and long-term change, implying more suitability of an area for wave energy resources assessment.

**Figure 6:**
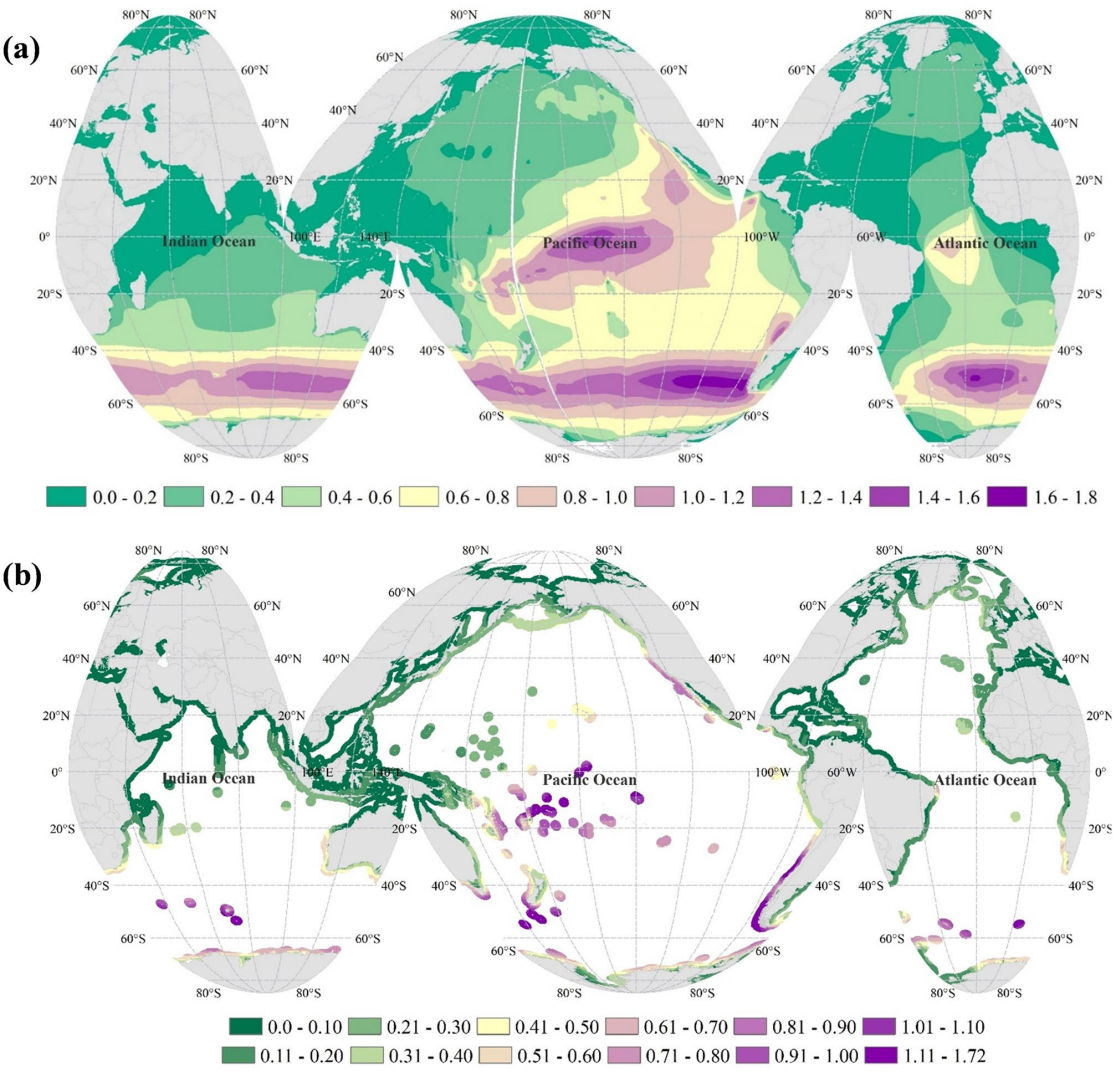
Sustainability Index (SI_p_) (**a**) globally and (**b**) in nearshore stripes. The figure has been generated using ArcGIS 10.2 and Natural Earth-Free vector and raster map data @ naturalearthdata.com.

Figure 6 shows SI_p_ values in the globe (Fig. 6a) and a nearshore stripe (Fig. 6b). Compared to the hotspots provided by Fairley et al.^53^ using only 10 years of ECMWF ERA5 wave data^54^ with the lack of the impact of changing climate and long-term variation of resources, our results provide more pronounced variability in the suitability of global coasts. According to Fig. 6, the southern hemisphere generally shows more suitability for wave energy extraction in terms of amount of energy and stability in both short and long term. This recognizes various islands in the southern Pacific, Atlantic and Indian Oceans, as well as coasts of Chile, southern coasts of New Zealand, southeast coasts of Australia, and western coasts of the United States and Mexico as the most suitable locations for wave energy extraction with SI_p_ values of higher than 0.8. The next suitable areas with SI_p_ values of 0.5–0.8 are south and southwest coasts of South Africa and Namibia, southwest of Australia, northwest of New Zealand, and eastern coasts of Papua New Guinea. The remaining areas in the northern hemisphere with more suitability based on SI_p_ are western and eastern coasts of Canada, east of Japan and Russia, west of Europe, Island, and south of Greenland.

## Discussion and conclusion

We used six decades of wind data for wave simulation with six-hourly temporal resolutions in time series of wave characteristics. The model was verified against both buoy measurements and satellite data. The six decades of wave data were initially divided by two time slices, i.e., Per_1 and Per_2, and the analysis revealed that the selection of the time slice affects the estimation of available wave energy due to the change in climate. This means that even a period of 30 years for resource assessment might not be sufficient. In addition, it showed the importance of assessing the long-term change in determining wave energy resources and hotspots. The results showed that the selection of different assessment periods, as defined in this study, can cause up to ± 25% difference in wave power resource assessment in deep waters. Moreover, the long-term change in wave power appears to be a function of swell wave height rather than a combination of swells and seas. This means that despite the wave power being directly calculated from significant wave height (which is the resultants of both wind sea and swells), the higher correlation between the change of wave power and swell height is correspondent to the low correlation between change of wave power and sea wave height.

The decadal variability analysis revealed that the change in wave power again follows that of the change in swell wave height. However, the change in wave climate has been different in different decades. For instance, the division of thirty yearly wave assessment shows a dominant decrease and increase in wave power in the second period in the northern and southern hemispheres, respectively. However, the decadal assessment shows various patterns in both the northern and southern hemispheres.

In order to quantify the relationship between the change of parameters, the RoC was calculated for wind and wave parameters in various decades in the entire output grid points, and its linear regression was obtained. The analysis showed a strong linear relationship between the RoC of P and H_swell_ in all decades with an average correlation coefficient of 0.91. On the other hand, the lowest correlation between RoC of P and wind and wave parameters was found to be with T_e,_ with an average correlation coefficient of 0.66.

Finally, the weighted arithmetic mean of the ratio of RoCs was calculated, and the RoC of wave power was found to be ~ 75 and 2.5 times the RoC of H_swell_ and WS with 91% and 85% accuracies, respectively. This means that the change in wave power can be predicted based on the change in swell wave height in the entire globe and even for the short periods of 10 years, with an accuracy of more than 90%. Moreover, based only on the wind characteristics, the change in wave power resources can be predicted with around 85% confidence.

Considering both short-term variation and long-term changes in the wave power, Sustainability Index (SI_p_) was utilized to detect the areas with the highest available wave power, lowest intra-annual fluctuations, and lowest long-term change in wave power. The classification based on SI_p_ revealed the priority areas mainly in the southern hemisphere, including the south and northwest of New Zealand, southeast and southwest of Australia, eastern coasts of Papua New Guinea, and south and southwest coasts of South Africa and Namibia. The Pacific islands and islands in the southern Indian Ocean are among the most suitable locations for wave energy extraction, with a high amount of wave power and low variability in terms of both monthly variation and long-term change. This is while the energy supply from non-renewable sources for such remote islands has been challenging considering the growing human population. The priority areas in the northern hemisphere are the west coasts of North America, western and eastern coasts of Canada, east of Japan and Russia, west of Europe, Iceland, and south of Greenland.

To summarize,

- It is essential to choose a suitable interval for wave energy resource assessment. Contrary to IEC’s recommendation for a minimum of 10 years for wave energy assessment, we showed that even with longer-term (e.g., 30 years) wave energy assessment, the change of assessment period can lead to an over/under-estimation of around 25% in wave power.
- The change in wave power correlates highly with the change in swell wave height rather than the significant wave height, and hence, it is possible to predict the change in wave power solely based on the predicted change in the swell climate.
- Considering the above-mentioned points, it is necessary to consider both short-term variation and long-term changes in selecting priority areas for energy extraction from the ocean waves.
- Such methodology for prioritizing the suitable areas for installing wave energy farms can be utilized from global to local scales.

To further expand this study, we aim to investigate the seasonal variability in the analysis to investigate the relationship between the change of seasonal wave energy and wind and wave climate as such a relationship might vary seasonally. Moreover, high-resolution analysis in priority areas will allow assessing the evolution of directional propagation of waves in nearshore areas and its impact on the sustainability of wave energy and, more specifically, the design of wave farms.

## Methods

### Model setup and data

Different sources are available for long-term wind and wave climate analysis, including satellite altimetry, in-situ measurements, and re-analysis data. Satellite data are often available globally, with low temporal resolution (e.g., daily), while in-situ measurements are only available in limited locations and various intervals despite having high temporal resolution. Re-analysis data prevail over such shortcomings with their global spatial coverage and higher temporal resolution (typically 1–6 h). There are various re-analysis wave data available with different spatio-temporal resolutions to be utilized for the purpose of trend analysis. In this study, we used JRA-55 wind data developed by the Japan Meteorological Agency (JMA)^55^, which is available from 1958 to date and enabled us to analyze six decades of wind and wave climate^25^. Using the re-analysis wind data, we simulated the wave characteristics for the period of availability of wind data (62 years) and analyzed the relationship between the wind and wave characteristics and the relationship between their change in the long-term on a global scale. Such assessment is independent of the source of wind data and concentrates on finding a relationship between the change of wind and wave climate on a global scale.

The re-analysis wind dataset of the JRA-55 model with the spatial and temporal resolutions of 60 km and 6 h, respectively, was used to force the numerical wave model i.e., SWAN (Simulating WAves Nearshore) Cycle III version 41.31^56^. Although SWAN has been developed to simulate the wave characteristics in nearshore, it has been successfully adopted to generate the oceanic wave climate as well^20,21,57^. The simulation was performed for the period of 1958–2019 and covered the whole globe. In order to reduce the computational effort and time for such a long-span simulation, the computational and output grid were considered with the spatial resolution of 1° from 0° to 359° in longitude and from 90° S to 90° N in latitude. Since ice data has not been considered as the inputs, the results are not discussed in the Arctic and Antarctica. The bathymetry information provided by the General Bathymetric Chart of the Oceans (GEBCO: https://www.gebco.net/) with 30 arc-sec spatial resolution was used to provide the bottom condition in the numerical model.

The computational grid with the frequency domain of 0.03–1 Hz with 36 bins on a logarithmic scale and the directional resolution of 10°, covering the whole globe (0° E–360° E in longitude and 90° S–90° N in latitude), was considered with a spatial resolution of 1 degree and computational time steps of 30 min^58^. The formulations of Komen et al.^59^ and Hasselmann et al.^60^ were used as source terms for the wind energy input and nonlinear 4-wave interaction (quadruplets), respectively. The formulation of Komen et al.^59^ was used for energy dissipation due to whitecapping, whereas the formulation of Hasselmann et al.^61^ was used for energy dissipation due to bottom friction. Calibration of the wave model has been done by tunning the whitecapping coefficient (C_ds2_), and the value of 2.96e−5 was selected based on trial and error in order to generate the least errors. The model outputs are the wave characteristics, including H_s_, H_swell,_ and wave energy period (T_m-10_ or T_e_)^62^.

SWAN directly generates the significant wave height (H_s_) and swell wave height (H_swell_) as HSIGN and HSWELL in the output parameters, respectively. It computes H_s_ (in meters) based on the following equation:

4 $$ {\text{H}}_{{\text{s}}} { = 4}\sqrt {{\iint }{{{\rm E}(\omega , \theta ){\rm d}\omega {\rm d}\theta }}} $$

where E(ω, θ) is the variance density spectrum and ω is the absolute radian frequency determined by the Doppler shifted dispersion relation. SWAN also computes H_swell_ based on the following formula, which is significant wave height associated with the low-frequency part of the spectrum, in meters, with ω_swell_ = 2πf_swell_ and f_swell_ = 0.1 Hz by default.

5 $$ {\text{H}}_{{{\text{swell}}}} { = 4}\sqrt {\mathop \int \limits_{{0}}^{{{\upomega }_{{{\text{swell}}}} }} \mathop \int \limits_{{0}}^{{{{2\pi }}}} {{{\rm E}(\omega , \theta ){\rm d}\omega {\rm d}\theta }}} $$

T_m-10_ is defined as m_-1_/m_0_, where m_n_ is the n-th moment of the energy density spectrum (E(f)) in which f is the frequency^56^ (Eq. 6):

6 $$ {\text{m}}_{{\text{n}}} { = }\mathop \int \limits_{{0}}^{\infty } {\text{f}}^{{\text{n}}} {\text{E}}\left( {\text{f}} \right){\text{df}} $$

Wave power is then calculated based on the deep water approximation formula (P ≈ 0.49 × H_s_^2^ × T_e_)^63^ in all grid points of the output domain covering the entire globe.

### Validation of the model

For a thorough and reliable examination of the possible uncertainties related to the wave hindcast accuracy, a detailed analysis of the error statistics of the utilized data was performed concerning long-term in-situ wave measurements and satellite data. We utilized two approaches in model validation to confirm the model's reliability for different parameters. The buoy measurements have high temporal resolutions, but they are only available in specific locations, while satellite data cover the whole globe, but with a lower temporal resolution for the wave data (typically daily). In addition, satellite wave measurements contain the wave height parameter solely. Hence, we first validated the model against the buoy measurements for wave height and wave period individually. Then, we utilized the satellite wave data to verify the model in the whole globe.

**Figure 7:**
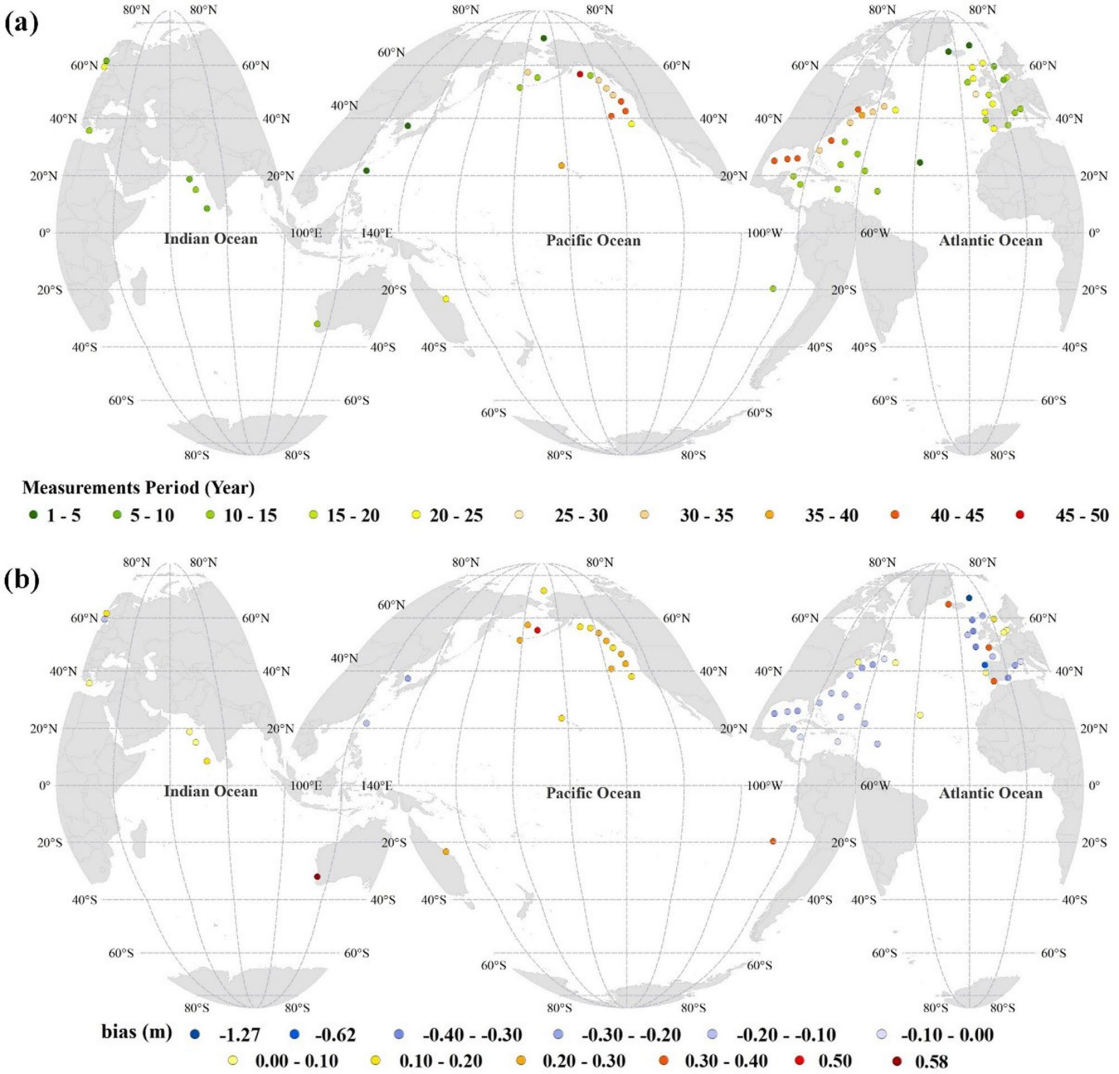
Map of (**a**) wave measurement period at each buoy location and (**b**) the bias in for H_s_. The figure has been generated using ArcGIS 10.2 and Natural Earth-Free vector and raster map data @ naturalearthdata.com.

The in-situ measurements were obtained from 64 buoys distributed worldwide with various recording periods (Fig. 7a). Since the study focuses on decadal-scale variability, we found it necessary to use the largest possible measurement period for the validation. Therefore, the used measurements cover the period of 1978–2019. Wave buoy measurements are provided by the Copernicus Marine Environment Monitoring Service (CMEMS) https://marine.copernicus.eu/ (last accessed 16.11.2020), and only offshore buoys are considered. Error indices, including the root means square errors (RMSE), scatter index (SI), bias, normalized bias (Nbias), and R were calculated for H_s_ and T_m02_ at each buoy location as follow:

7 $$ {\text{RMSE}}\; = \;\sqrt {\frac{{1}}{{\text{N}}}\mathop \sum \limits_{{\text{i = 1}}}^{{\text{N}}} \left( {{\text{P}}_{{\text{i}}} - {\text{M}}_{{\text{i}}} } \right)^{{2}} } $$

8 $$ {\text{SI}}\; = \;\frac{{{\text{RMSE}}}}{{\frac{{1}}{{\text{N}}}\mathop \sum \nolimits_{{\text{i = 1}}}^{{\text{N}}} {\text{M}}_{{\text{i}}} }} $$

9 $$ {\text{bias}}\;{ = }\;\mathop \sum \limits_{{\text{i = 1}}}^{{\text{N}}} \frac{{1}}{{\text{N}}}{ }\left( {{\text{P}}_{{\text{i}}} - {\text{M}}_{{\text{i}}} } \right) $$

10 $$ {\text{Nbias}}\;{ = }\;\frac{{1}}{{\frac{{1}}{{\text{N}}}\mathop \sum \nolimits_{{\text{i = 1}}}^{{\text{N}}} {\text{(M}}_{{\text{i}}} {) }}}\mathop \sum \limits_{{\text{i = 1}}}^{{\text{N}}} \frac{{1}}{{\text{N}}}{\text{ (P}}_{{\text{i}}} - {\text{M}}_{{\text{i}}} {)} $$

where M_i_ is the measured value, P_i_ is the predicted value, and N is the number of data. Calculation of R for the model’s outputs has been done using Eq. (1), where $\stackrel{\mathrm{-}}{\text{x}}$ and $\stackrel{\mathrm{-}}{\text{y}}$ are the measured and modeled values, respectively.

For assessing the model’s accuracy, spatially, the H_s_ biases at each buoy location are mapped in Fig. 7b. Figure 7b shows that the wave model performance varies at the spatial scale in terms of bias for H_s_. The wave model slightly overestimates H_s_ in the North Pacific and Indian Oceans and slightly underestimates it in the North Atlantic Ocean. Since the wave model was only forced by the wind fields and bathymetry data, the spatial differences in the wave model biases may be related to the wind climate regime and/or the accuracy of the wind field and sea surface ice. However, the absolute bias for H_s_ at most buoy locations does not exceed 30 cm. The summary of error statistics presented in Table 1 also reflects a suitable performance of the SWAN model. The average correlation coefficient is 0.89 for H_s_ and 0.72 for the mean periods (T_m02_). Thus, the average scatter index equals 0.29 and 0.21 for H_s_ and T_m02_. These results reflect the accuracy of the used model on a global scale. In addition, as mentioned in the previous section, ice data are not taken into account during the wave simulation, and the largest errors are observed in the far North Atlantic buoy (Fig. 7b).

**Table 1:** Summary of error statistics in the estimated H_s_ and mean periods determined for 64 buoy locations.

	H_s_					T_m02_					Distance from the closest grid point (°)
	R	SI	bias (m)	N.Bias	RMSE (m)	R	SI	bias (s)	N.Bias	RMSE (s)	
Lowest	0.81	0.17	0.02	− 0.51	0.27	0.51	0.13	0.01	− 0.41	0.69	0.00
Mean	0.89	0.29	0.22	− 0.03	0.58	0.72	0.21	0.67	− 0.08	1.24	0.40
Largest	0.95	0.46	− 1.27	0.24	1.73	0.83	0.40	− 2.59	0.11	3.12	0.69

**Figure 8:**
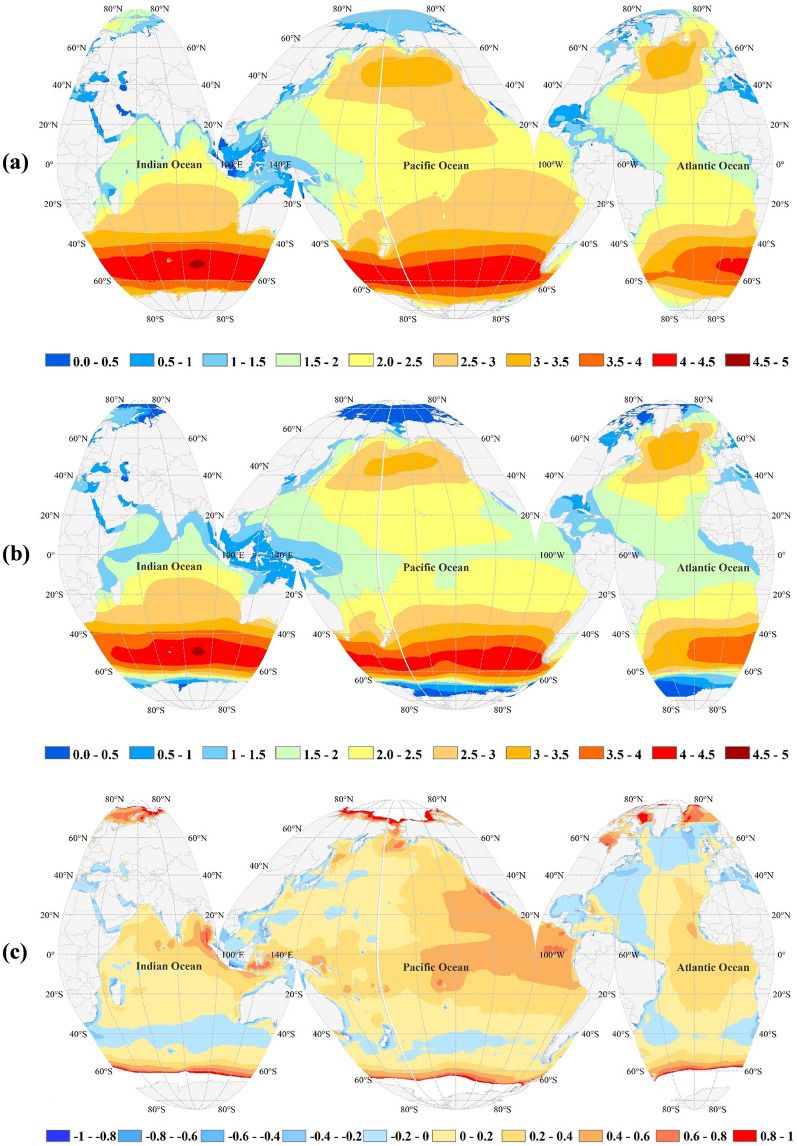
(**a**) mean annual H_s_ (m) based on SWAN output (**b**) mean annual H_s_ (m) based on satellite altimetry, and (**c**) model bias (m). The figure has been generated using ArcGIS 10.2 and Natural Earth-Free vector and raster map data @ naturalearthdata.com.

For satellite altimetry, the near real-time gridded wave data (1° × 1°, regular grid) with a daily temporal resolution were used. These data are provided by Aviso (https://www.aviso.altimetry.fr/). Figure 8 shows the spatially distributed mean annual H_s_ and bias for modeled H_s_ against satellite observation. According to Fig. 8, the bias is limited to ± 20 cm over a large part of the globe. The bias values are slightly larger in the eastern Pacific Ocean and the northern Indian Ocean. A probable overestimation of the quantitative results (e.g., wave powers) estimated in these regions should be carefully considered in this study. Nevertheless, these biases do not affect the qualitative findings or raised conclusions.

## Data Availability

The input wind data are available from the developer’s webpage: https://jra.kishou.go.jp/JRA-55/index_en.html. The wave model outputs are available at: https://bit.ly/3LquZ3h.
